# Supplementary material for: Exploring the Bio-Functional Effect of Single Nucleotide Polymorphisms in the Promoter Region of the TNFSF4, CD28, and PDCD1 Genes
Source: J Clin Med. 2023 Mar 10;12(6):2157. doi: 10.3390/jcm12062157 (PMC10058121; doi:10.3390/jcm12062157)
Supplement: Supplementary file 1 [file jcm-12-02157-s001.zip › Supplementary Table S1.pdf]

Supplementary Table S1. The RLU of *TNFSF4* promoter reporter assay (16 independent tests).

TNFSF4															F		83.255	p^a	<0.001
																	Mean	SD	p^b
TNFSF4 wild type	1.00	1.00	1.00	1.00	1.00	1.00	1.00	1.00	1.00	1.00	1.00	1.00	1.00	1.00	1.00	1.00	1.00	0.00	
rs1234314 C>G	0.17	0.48	0.45	0.36	0.44	0.29	0.35	0.21	0.39	0.32	0.29	0.32	0.26	0.27	0.24	0.27	0.32	0.09	0.003
rs45454293C>T	3.94	4.63		3.84		4.44	4.38	5.83	5.92	4.94	3.03	6.17	3.75	4.48	5.39	4.04	4.63	0.92	<0.001

p^a: the p value of ANOVA analysis; p^b: the p value of post hot test; SD: standard deviation. The blank was indicated that the data was outlier (not within 2 standard deviation).
