# Supplementary material for: Exploring the Bio-Functional Effect of Single Nucleotide Polymorphisms in the Promoter Region of the TNFSF4, CD28, and PDCD1 Genes
Source: J Clin Med. 2023 Mar 10;12(6):2157. doi: 10.3390/jcm12062157 (PMC10058121; doi:10.3390/jcm12062157)
Supplement: Supplementary file 1 [file jcm-12-02157-s001.zip › Supplementary Table S2.pdf]

Supplementary Table S2. The RLU of *PDCD1* promoter reporter assay (11 independent tests).

**PDCD1**												**F**	**70.093**	**p^a**	**<0.001**
													Mean	SD	p^b
PDCD1 wild type	1.00	1.00	1.00	1.00	1.00	1.00	1.00	1.00	1.00	1.00	1.00	1.00	1.00	0.00	
rs5839828 G>del	1.14	1.53	1.09	1.55	1.67	1.81	1.46	1.23	1.44	1.23	1.1	1.19	1.37	0.24	<0.001
rs36084323 C>T	0.77	0.73	0.61	0.58	0.62	0.62		0.78	0.73	0.78	0.67	0.62	0.68	0.07	<0.001

p^a: the p value of ANOVA analysis; p^b: the p value of post hot test; SD: standard deviation. The blank was indicated that the data was outlier (not within 2 standard deviation).
