# Supplementary material for: Exploring the Bio-Functional Effect of Single Nucleotide Polymorphisms in the Promoter Region of the TNFSF4, CD28, and PDCD1 Genes
Source: J Clin Med. 2023 Mar 10;12(6):2157. doi: 10.3390/jcm12062157 (PMC10058121; doi:10.3390/jcm12062157)
Supplement: Supplementary file 1 [file jcm-12-02157-s001.zip › Supplementary Table S3.pdf]

Supplementary Table S3. The RLU of *CD28* promoter reporter assay (15 independent tests).

CD28														F	0.644		p^a	0.633
																Mean	SD	p^b value
CD28 wild type	1.00	1.00	1.00	1.00	1.00	1.00	1.00	1.00	1.00	1.00	1.00	1.00	1.00	1.00	1.00	1.00	0.00	
rs28541784T>C	1.31	1.00	0.94	0.81	1.47	0.77	0.37	0.31	0.97	1.07	0.88		0.91	0.96	1.11	0.92	0.31	0.850
rs200353921A>T	1.34	1.25	0.86	0.65	0.98	1.11	0.85		0.97	0.87	1.20	0.92	1.00	1.01	0.88	0.99	0.18	1.000
rs3181096C>T	0.74	0.79	0.97	0.90	0.89	0.69	1.17	1.00	0.65	0.63		1.13	0.82	1.27	0.91	0.90	0.20	0.694
rs3181098G>A	0.97	1.26	0.69	1.10	1.16	0.71	0.57	1.40		0.88	0.93	1.32	0.73	1.03	0.85	0.97	0.25	0.996

p^a: the p value of ANOVA analysis; p^b: the p value of post hot test; SD: standard deviation. The blank was indicated that the data was outlier (not within 2 standard deviation).
